# On the diverse utility of Cu doped ZnS/Fe_3_O_4_ nanocomposites

**DOI:** 10.1038/s41598-024-62611-0

**Published:** 2024-05-22

**Authors:** Shirin Kalantari, Ali Shokuhfar

**Affiliations:** Advanced Materials and Nanotechnology Research Laboratory, Faculty of Materials Science and Engineering, K. N. Toosi University of Technology, Tehran, Iran

**Keywords:** Photocatalyst, Sonochemical, Composite, Nanoscale materials, Photocatalysis, Synthesis and processing

## Abstract

The global water crisis is a growing concern, with water pollution from organic dyes being a significant issue. Photocatalysis has emerged as a sustainable and renewable method for removing organic pollutants from wastewater. The study synthesized innovative (2.5, 5 and 10 wt%) Cu doped zinc sulfide/iron oxide nanocomposites using a sonochemical method, which have versatile applications in adsorption and photocatalytic degradation of organic pollutants in wastewater. The nanocomposites underwent comprehensive characterization using powder X-ray diffraction, fourier-transform infrared spectroscopy, photoluminescence spectroscopy, Ultraviolet–Visible spectrophotometer, field emission scanning electron microscopy combined with energy dispersive X-ray spectroscopy and Mott–Schottky analysis. The synthesized samples demonstrate strong adsorption ability to remove RhB and MB dyes. Afterward, we evaluated their capability to degrade Rhodamine B (RhB) dye under UV light exposure. The greatest photocatalytic efficiency was noticed when employing a UV-C lamp in combination with the 10 wt% Cu doped ZnS/Fe_3_O_4_ nanocomposite as photocatalyst (98.8% degradation after 60 min irradiation). The Langmuir–Hinshelwood model can be used to describe the pseudo first order kinetics of RhB dye photodegradation. The calculated ban gap values are 4.77, 4.67, and 4.55 eV, for (2.5, 5 and 10 wt%) Cu doped ZnS/Fe_3_O_4_, respectively. Furthermore, 10 wt% Cu doped ZnS/Fe_3_O_4_ showed good recyclability, with a degradation rate of 89% even after five cycles. Consequently, prepared samples have outstanding photocatalytic activity and can be used as useful adsorbents in water purification.

## Introduction

The escalating pace of industrialization has led to a growing and severe issue of water contamination on a global scale. According to statistical forecasts, by the year 2025, more than one billion individuals residing in arid regions will undoubtedly face water scarcity^1,2^. Industrial wastewater discharges constitute the primary cause of water contamination, comprising organic dyes, heavy metals, oil, and microbial pollutants^3^. Among them, major compounds that harm the aquatic ecology are dye molecules^4,5^. Notably, they exhibit carcinogenic properties and result in invertebrate mutations^4^. Consequently, the detoxification of these pollutants has become a significant global concern, prompting the exploration of numerous physical (adsorption and ion exchange), chemical (Photolysis, electrochemical), biological, AOPs (photocatalytic and sono catalytic), and hybrid approaches for water treatment^6–8^. The positive aspects of physical, chemical, and biological approach are straightforward, adaptable, effective, and environmentally benign. Additionally, it has several drawbacks, including high expenses, a problem with the disposal of secondary sludge, and excessive energy consumption^4,6,9,10^. On the other hand, advanced oxidation processes are already widely used because of their potential for eliminating dye from wastewater. Significant advantages of AOPs include quick dye removal, affordability, environmental friendliness, efficacy against persistent pollutants, reduced time requirements, and high output^6^. Adsorption and catalysis technique is currently being looked into for the photodegradation of pollutants, which was considered to be one of the most effective approaches^3,11–13^. To date, researchers have determined numerous semiconductors as appropriate photocatalysts for removing hazardous pollutants from water using a variety of elimination procedures and proclaimed them effective^14–16^.

Zinc sulfide (ZnS), which has improved electrical mobility, water insolubility, thermal endurance, toxicity-free and affordable, is one of the most pledging semiconductors^17,18^. With a broad bandgap of 3.68 eV and a suitable negative redox potential of the conduction band (− 1.36 eV) and valence band (+ 2.35 eV), ZnS is an n-type semiconductor photocatalyst that possesses outstanding chemical stability towards oxidation and hydrolysis. In a tetrahedral structure, ZnS can be found in a variety of morphologies, including cubic (sphalerite) and hexagonal (wurtzite)^17,19^. The majority of semiconductors, like ZnS, have a significant bandgap, which has restricted their photoabsorption in the ultraviolet or close to ultraviolet areas. Different modification techniques, including as dye sensitization, coupling, doping, and capping, have so far resulted in a reduced band gap and a greater duration of electron and hole recombination, allowing for the control of organic reactions under mild and visible-light environment^20^. In order to customize the band structure, a number of researchers investigated the impact of doping transition metal (Zr, Pd, Mn, and Cu) ions on photocatalytic performance of ZnS^21–25^.

Recycling of nanocomposites is essential for long-term tracking and treatment of the environment. Centrifugation, filtration, and sedimentation are the most popular ways for separating, however these techniques are arduous, require considerable time, and result in the loss of functional qualities. The magnetic separation process offers the advantages of being straightforward to use and saving time. Superparamagnetic Fe_3_O_4_ nanoparticles, with enormous specific surface area and strong saturation magnetization, have proven to be the best option for magnetic supports^26^. Some investigations on the magnetic separation usage of Fe_3_O_4_ have been undertaken^27–32^.

Shi et al. established a mild, economical, and safe technique for manufacturing perpendicularly oriented MnO_2_ nanosheets coated on Fe_3_O_4_ fibers using PDA as a platform that enhances adsorption efficacy while also supporting magnetic separation^30^. Saha et al. have previously explained the process of synthesizing phosphoramidate functionalized Ag coated citrate-Fe_3_O_4_ nanoparticles. The primary aim of this endeavor was to uphold a moderate level of magnetic responsivity of the nanoparticles, thereby facilitating their separation through the utilization of conventional magnets^31^. Kim et al. reported the fabrication of Fe_3_O_4_ nanoparticles@graphene-poly-*N*-phenylglycine nanofibers to efficiently recyclable^33^.

Several notable studies have been published on the incorporation of Cu as a dopant. For instance, Karthik et al. published a study on the environmentally friendly production of Cu-doped ZnO nanoparticles. These nanoparticles have promising potential in the photocatalytic degradation of harmful organic pollutants, such as Methylene blue (MB), Indigo Carmine (IC), and Rhodamine B (RhB)^34^. Also, Wang et al. reported synthesis of Cu-doped Bi_2_MoO_6_ microflower to enhance efficiency in the process of photocatalytic nitrogen fixation. The results of the photocatalytic experiment demonstrated a substantial improvement in the catalytic performance of Bi_2_MoO_6_ for the reduction reaction of N_2_, which can be attributed to the introduction of Cu doping^35^. The synthesis of Cu-doped biphasic Bi_2_O_3_ samples with the hydrothermal method and the surface modification by Ni, Pt, and Pd were reported by Sharma et al. The photocatalytic application of these samples was assessed by testing their performance against RhB. Finally, the samples with Pd demonstrated the most significant degradation of RhB, achieving complete degradation of the dye in 50 min^36^. Another study published by Sharma et al. on the development of metal ion (M = Co^+2^, Ni^+2^, and Cu^+2^) doped-In_2_O_3_ photocatalysts using a straightforward solution combustion method. The synthesized compounds were evaluated for degradation of RhB and TC^37^.

Various techniques, such as hydrothermal, solvothermal, ball milling, magnetron sputtering, chemical vapor deposition, solid-state reaction, sol–gel methods, photoreduction, and spray pyrolysis, have been employed to synthesize nanostructures. However, these methods often entail high costs, energy consumption, and prolonged operational durations. Seeking more efficient approaches for photocatalysis, sonochemical synthesis has emerged as a contemporary method that utilizes sound waves to convert reactant materials into nanostructures with excellent photocatalytic performance. Sound waves, generated by a vibrating object, act as sources of mechanical energy and pressure in this process. Sonochemical synthesis is recognized for its economic efficiency, reducing the consumption of extra reagents and eliminating the need for additional heat treatment. The method yields high surface area nanostructures with minimal agglomeration, as compared to heat treatment, which may reduce surface area. In sonosynthetic chemistry, sound waves activate precursor materials, making it a low-power procedure that significantly reduces precipitation time for various semiconductors. Moreover, sonochemical synthesis results in the synthesis of materials with homogeneous and consistent morphology, making it a promising technique in the realm of green chemistry^38^.

Our research team was motivated by the aforementioned concepts to synthesize Cu doped ZnS/Fe_3_O_4_ nanocomposites using a swift and successful sonochemical method. This multipurpose nanocomposite could be used as an adsorbent and photocatalyst to remove and degrade organic contaminants. Under UV light, the efficacy of these nanocomposites for photocatalytic degradation of dye molecules (RhB) and reaction kinetics were examined. Furthermore, the recycling ability of the manufactured nanocomposite was investigated via its magnetic separation characteristic. The 10 wt% Cu doped ZnS/Fe_3_O_4_ nanocomposite displayed excellent photostability and reusability after five following cycles.

## Materials and methods

### Materials

Chemical materials of the analytical grade were utilized in this study and were acquired from Sigma-Aldrich and Merck chemicals. All reagents were used directly from the supplier without any additional purifying steps. Table 1 shows chemical materials with purity percentage.

**Table 1:** Chemical materials that used in synthesis process of Cu doped ZnS/Fe_3_O_4_.

Materials	Chemical formula	Purity (%)	Supplier
Ferric chloride hexahydrate	FeCl_3_⋅6H_2_O	99	Merck
Ferrous chloride tetrahydrate	FeCl_2_⋅4H_2_O	≥ 98.0	Merck
Ammonia solution	NH_3_	25	Merck
Hydrochloric acid	HCl	37	Merck
Sodium sulfide hydrate	Na_2_S⋅xH_2_O	≥ 60	Sigma-Aldrich
Zinc nitrate hexahydrate	Zn (NO_3_)_2_⋅6H_2_O	98	Sigma-Aldrich
Copper(II) nitrate hemi (pentahydrate)	Cu(NO_3_)_2_⋅2.5H_2_O	98	Merck
Dodecyl sulfate sodium salt	C_12_H_25_NaO_4_S	≥ 95	Merck
Ethanol	C_2_H_5_OH	≥ 99.9	Merck
Rhodamine B	C_28_H_31_ClN_2_O_3_	≥ 80	Merck
Methylene blue	C_16_H_18_N_3_SCl	≥ 82	Merck

### Preparation of Fe_3_O_4_ and Cu doped ZnS/Fe_3_O_4_

Referring to the article^39^, the co-precipitation method was employed to synthesize Fe_3_O_4_ nanoparticles. Initially, a mixture of ferrous chloride and ferric chloride in a 1:2 M ratio was prepared in 100 mL of deionized water. The solution was stirred at 60 °C for 0.5 h. Subsequently, dropwise addition of ammonia solution, while continuously stirring, was carried out until the pH of the solution approached 10. A black precipitate was generated and maintained at 80 °C for 2 h. Then, the magnetite was recovered from the solution with a strong magnet and repeatedly rinsed with deionized water. So it was dried at 100 °C for 12 h.

**Figure 1:**
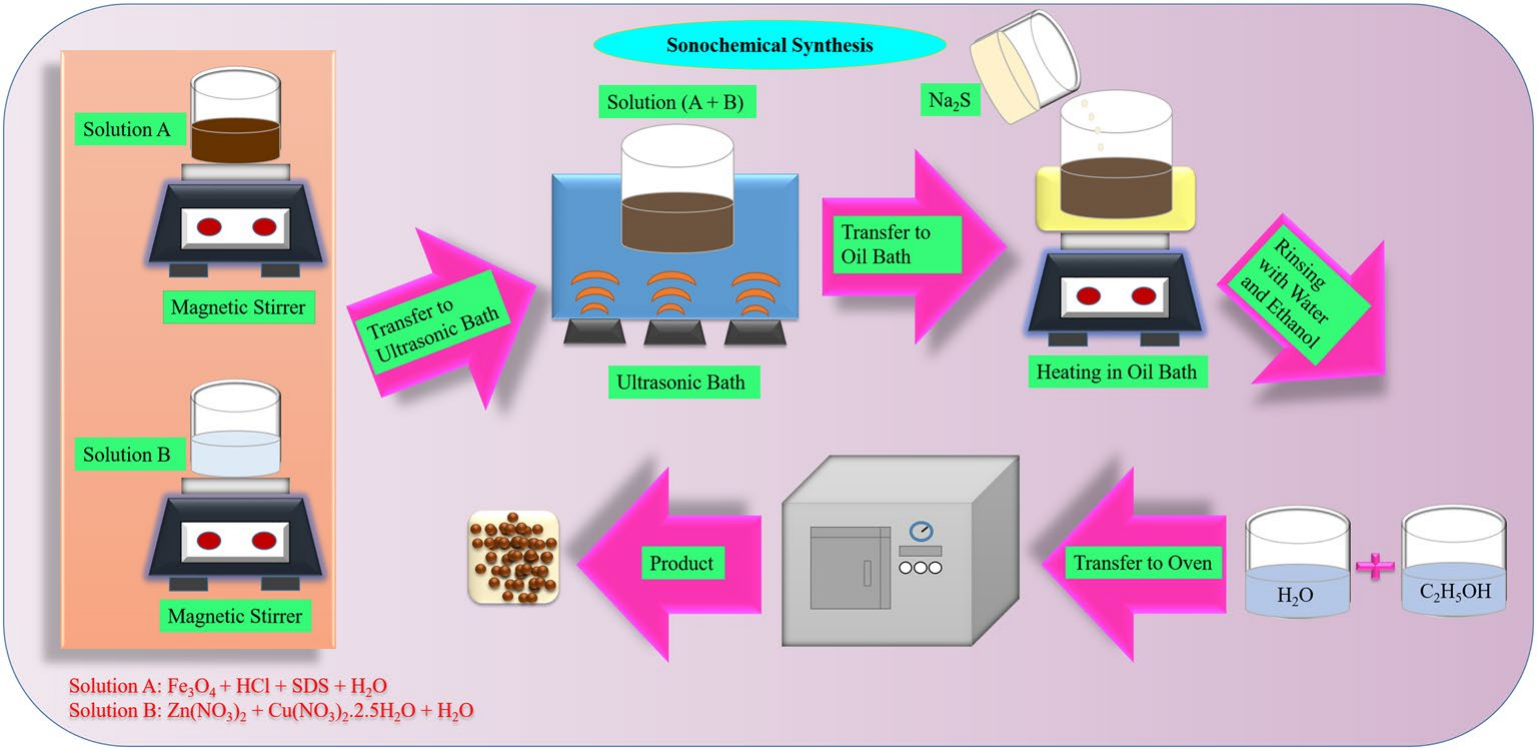
Synthesis of Cu doped ZnS/Fe_3_O_4_ nanocomposite.

Cu doped ZnS/Fe_3_O_4_ nanocomposites were generated using a sonochemical method (Fig. 1). At first, HCl (100 mL; 0.05 mol L^−1^) and Fe_3_O_4_ (0.4 g) were combined and sonicated for 30 min. The particles were gathered using a magnet and purified repeatedly with deionized water. Additionally, Fe_3_O_4_ particles were added to sodium dodecyl sulfate (SDS) aqueous solution (100 mL; 0.1 mol L^−1^) to create functionalized Fe_3_O_4_ using anionic surfactant. The combination was exposed to ultrasonic for 20 min. Furthermore, modified Fe_3_O_4_ was magnetically extracted and added to 60 mL of Zn(NO_3_)_2_ and Cu(NO_3_)_2_⋅2.5H_2_O (2.5, 5, and 10wt% aqueous solution, 0.1 mol L^−1^). After 20 min of sonication, the mixture was put in a bath with oil at 60 °C. Finally, an aqueous solution of Na_2_S (60 mL; 0.1 mol L^−1^) was added to the mixture dropwise. After agitating for 2 h, the precipitate was separated with a magnet, rinsed with water and ethanol and dried in an oven at 60 °C for overnight.

### Characterization

X-ray diffraction (XRD) measurements were conducted utilizing the Bruker D8 ADVANCE X-RAY DIFFRACTOMETER, employing Cu-Kα radiation (λ = 0.15418 nm). The Fourier transform infrared (FTIR) spectra were obtained using a PerkinElmer spectrometer. A field-emission scanning electron microscope (FESEM, MIRA3, TESCAN) was used to investigate the morphology of the produced samples. The elemental composition of the samples was assessed through energy dispersive spectroscopy analysis (EDS). Photoluminescence (PL) spectra were collected using an Avaspec 2048 TEC spectrometer, covering a range from 200 to 800 nm (excitation wavelength: 200 nm). The UV-1100 UV/Vis spectrophotometer (220V, 50Hz) was used to examine the UV–Vis absorption spectra of the samples. Mott-Schottky measurements were used to evaluate the electronic structure inquiry. M-S analysis was performed at a frequency of 0.5 kHz using a three-electrode system (glassy carbon, platinum disk, and Ag/AgCl for the working electrode, counter electrode, and reference electrode, respectively) in an aqueous electrolyte containing 0.1 M Na_2_SO_4_.

### Adsorption and photocatalysis experiments

The adsorption of MB and RhB molecules was accomplished by dissolving 100 mg of (2.5, 5, and 10 wt%) Cu doped ZnS/Fe_3_O_4_ in 100 mL aqueous dye solution (with a concentration of 10^–5^ M) at room temperature under the dark and UV light condition, respectively. Centrifugation was used to separate the (2.5, 5, and 10 wt%) Cu doped ZnS/Fe_3_O_4_ after a given amount of time. The equilibrium dye concentrations were determined using the UV–Vis adsorption spectra. This evaluation was performed at the calibrated maximum wavelength of 668 nm and 554 nm for MB and RhB, respectively. Equation (1) was used to compute the adsorption capacity of Cu doped ZnS/Fe_3_O_4_^40,41^.

1 $${q}_{t}=\frac{{C}_{0}-{C}_{t}}{m}V.$$

C_0_ (mg L^−1^) and C_t_ (mg L^−1^) represent the primary and instantaneous (t (h)) content of the dye solution, respectively. m (g) denotes the weight of the employed adsorbent, while V (L) corresponds to the volume of solution.

For photodegradation, the UV lamp with a wavelength of 254 nm, was positioned at a distance of 26 cm from the beaker. Following exposure within a designated time period, the degree of color removal and, as a result, the effectiveness of dye photodegradation will be determined by the decrease in absorbance spectra of the samples at the respective maximum absorption wavelengths of the dyes.

The calculation of the degradation efficiency (%D) was performed according to the given Eq. (2)^42^.

2 $$\text{\%D}=\frac{{\text{C}}_{0}-\text{C}}{{\text{C}}_{0}}\times 100.$$

The dye concentrations before and after irradiation for a predetermined amount of time are shown by the letters C_o_ and C, respectively. To investigate the durability and repetition of the 10 wt% Cu doped ZnS/Fe_3_O_4_ photocatalyst, RhB dye photodegradation recycling studies were done, with identical experimental settings for five cycles. The photocatalysts were isolated from the aqueous solution after each cycle through the application of an external magnet. Subsequently, cleansed using water and acetone, before dried. The assessment of the degradation efficiency of each cycle was conducted to ascertain the recyclability of the photocatalyst.

## Results and discussion

### X-ray diffraction (XRD) study

**Figure 2:**
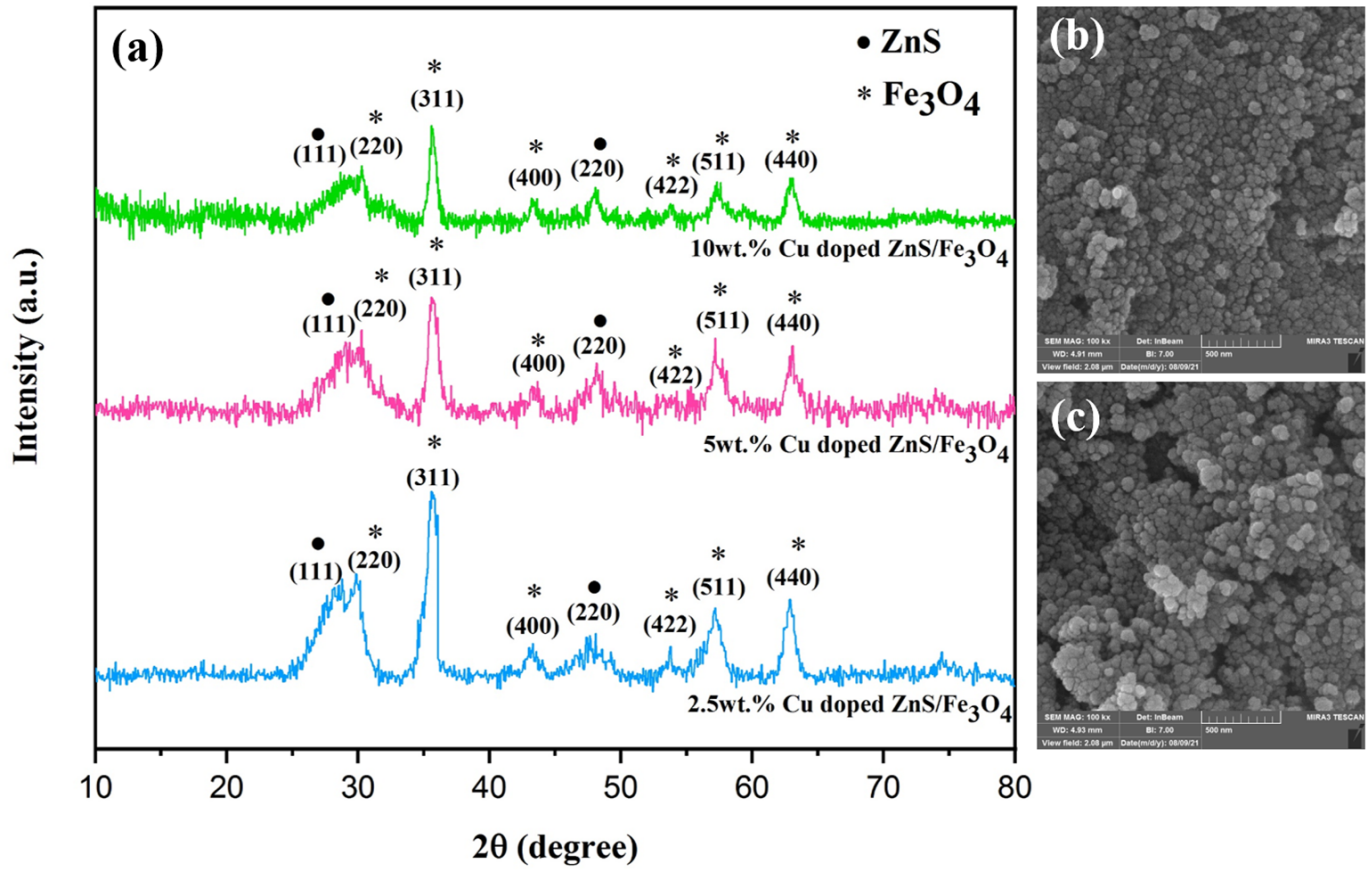
(**a**) XRD pattern of (2.5, 5 and 10 wt%) Cu doped ZnS/Fe_3_O_4_ nanocomposites and FESEM images of (**b**) 5 wt% Cu doped ZnS/Fe_3_O_4_ and (**c**) 10 wt% Cu doped ZnS/Fe_3_O_4_ nanocomposites.

The XRD pattern of (2.5, 5 and 10 wt%) Cu doped ZnS/Fe_3_O_4_ nanocomposites displayed in Fig. 2a. As stated in our prior investigation, the XRD patterns of the synthesized samples exhibit discernible peaks corresponding to magnetite and ZnS, while no peaks indicative of impurity can be identified, and therefore the inclusion of dopants does not affect the phase structure of ZnS/Fe_3_O_4_, and the Cu ions are effectively integrated into the lattice structure of ZnS without forming CuS, as mentioned in the literatures^43,44^. Substitution doping is deemed beneficial when the two elements possess equivalent ionic radii. It is worth noting that Cu^2+^ and Zn^2+^ have similar ionic radii, with values of 0.73 and 0.74 Å, respectively. So Cu^2+^ can easily substitute with Zn^2+^ in the ZnS lattices, compared to S^2−^^45^. The FE-SEM images of the (5 and 10 wt%) Cu-doped ZnS/Fe_3_O_4_ nanocomposites (Fig. 2b,c) show a homogeneous and symmetrical spherical shape, with some agglomeration. The mean size of the crystallite (D) in all samples was determined using Scherrer’s formula (Eq. 3)^46,47^:

3 $$\text{D}=\frac{0.9\lambda }{\beta \text{cos}\theta }.$$

Table 2 presents the average crystallite size of Fe_3_O_4_ and ZnS as determined through the implementation of the Scherrer technique on the peaks exhibiting considerable intensity. It is evident that the size of the crystallites in each nanocomposite varies from 12 to 58 nm, thereby confirming their nano-scale nature.

**Table 2:** The average crystallite size (nm) ascertained through XRD.

Sample	Fe_3_O_4_ (311)	Fe_3_O_4_ (400)	ZnS (111)	ZnS (220)
2.5wt% Cu doped ZnS/Fe_3_O_4_	33	12	14	58
5wt% Cu doped ZnS/Fe_3_O_4_	28	14	10	17
10wt% Cu doped ZnS/Fe_3_O_4_	20	23	23	14

## Fourier-transform infrared (FTIR) study

**Figure 3:**
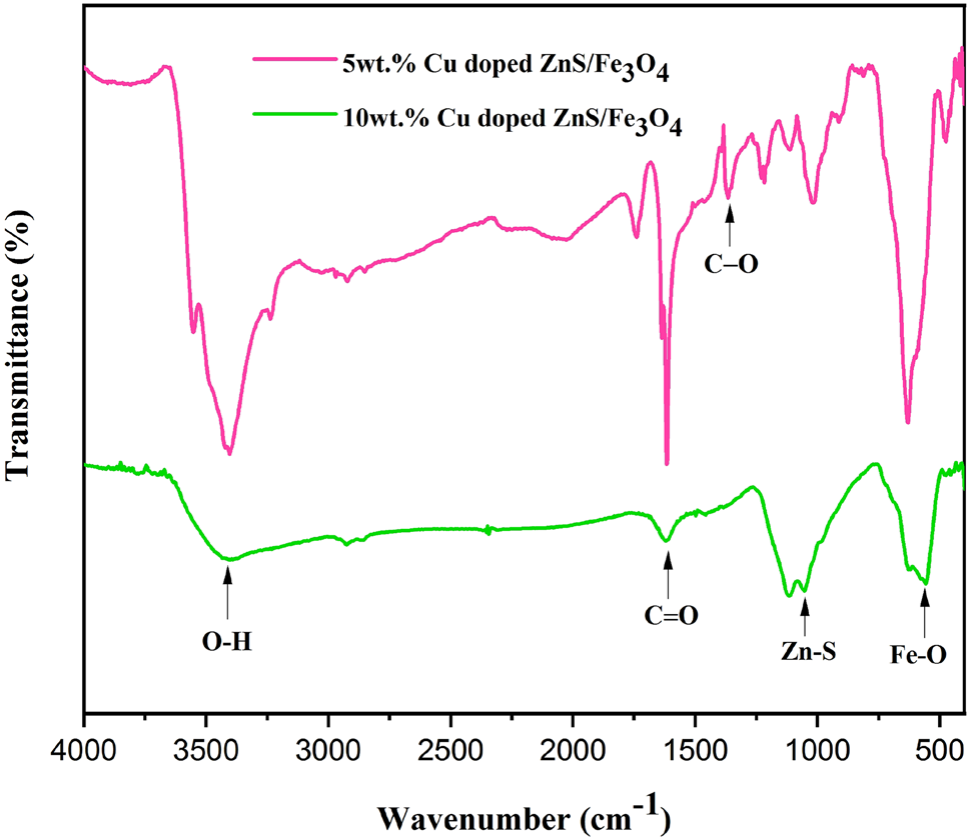
FTIR spectra of (5 and 10 wt%) Cu doped ZnS/Fe_3_O_4_ nanocomposites.

Figure 3 exhibits the FTIR spectra of (5 and 10 wt%) Cu doped ZnS/Fe_3_O_4_ nanocomposites. As documented in various literary works, the absorption bands observed at 1622.75 cm^−1^ and 3435.29 cm^−1^ can be ascribed to the stretching vibrations of C=O and O–H, respectively^43^. In 5 wt% Cu doped ZnS/Fe_3_O_4_ nanocomposite, the Fe–O bond stretching vibrations are also associated with the notable peaks of absorption at 629.72 cm^−1^ and 556.76 cm^−1^, indicating a properly formed Fe_3_O_4_ structure^48^. Also, the stretching vibration of Zn–S is represented by the peak at 1013 cm^−1^. The peaks observed at 1305.06 cm^−1^ is ascribed to the presence of C–O bond^39^. Furthermore, the lack of any absorption band associated with CuS suggests that Cu is present in a pure state. Notably, there has been no occurrence of any form of chemical bonding between Cu and the nanocomposite. According to the current investigation, the substitution of transition metals in the crystal structure of ZnS nanoparticles has been adequately accomplished. The allocation of peaks for (5 and 10 wt%) Cu-doped ZnS/Fe_3_O_4_ is presented in Table 3.

**Table 3:** Peaks assignment of (5 and 10 wt%) Cu doped ZnS/Fe_3_O_4_.

Assignments	Cu (5%)	Cu (10%)
Fe–O stretching vibrations	629.72, 556.76	625.46, 557.08
Zn–S stretching vibrations	1013	1120.28

### Photoluminescence spectroscopy (PL) study

**Figure 4:**
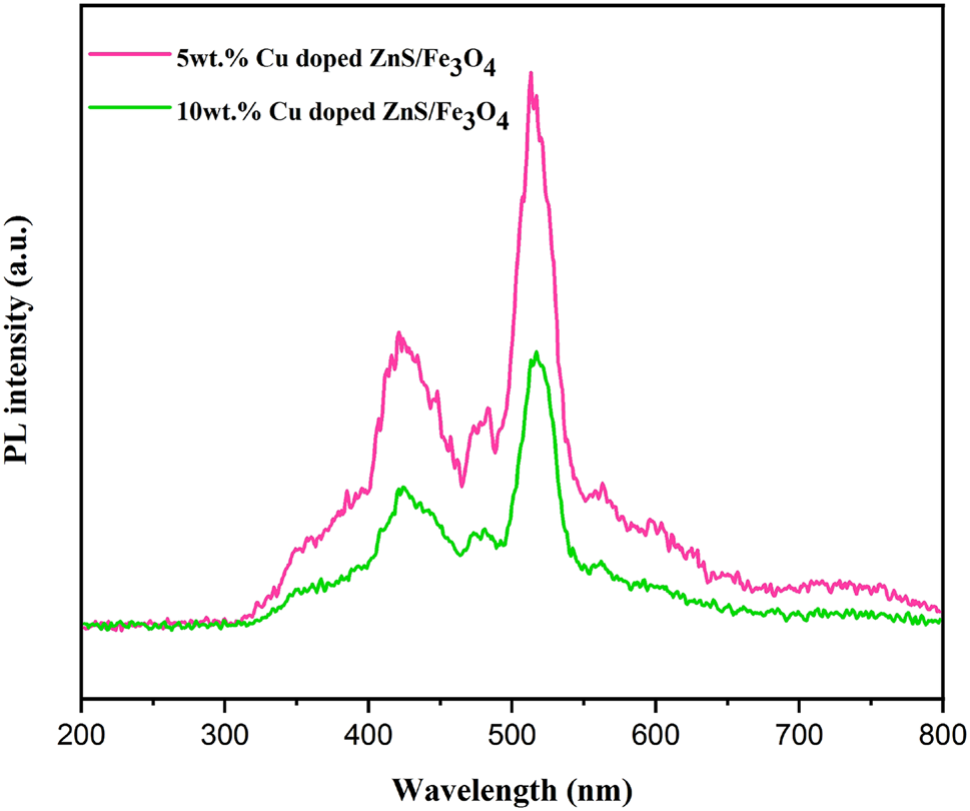
Photoluminescence emission spectra of (5 and 10 wt%) Cu doped ZnS/Fe_3_O_4_ nanocomposites.

To investigate the optical characteristics of (5 and 10 wt%) Cu doped ZnS/Fe_3_O_4_ nanocomposites, photoluminescence spectroscopy, as illustrated in Fig. 4, was performed with an excitation wavelength of 200 nm and a range of 200–800 nm. The PL emissions of samples are at approximately 423, 480, and 516 nm, which maximum emission was measured at 516 and 423 nm, although a faint emission peak was recorded at 480 nm. The recombination of electrons from a shallow state to the sulfur vacancies causes the emission peak at 423 nm. Zinc vacancies in the ZnS lattice are also responsible for the emission peak at 480 nm^49^. Fundamentally, the samples emit light at these specific wavelengths due to the presence of different types of vacancies in the material, with sulfur and zinc vacancies contributing to the observed emissions at 423 and 480 nm, respectively. The PL intensities of 10 wt% Cu doped photocatalysts were lower than those of 5 wt% Cu doped ZnS/Fe_3_O_4_. The intensity of this PL spectrum falls as the weight percent of dopant increases. Photo-generated e^−^/h^+^ pair recombination decreases as intensity decreases. This proves that photocatalytic efficiency is influenced by PL results. Higher photocatalytic activity is found in photocatalysts with decreased exciton recombination^50^. Table 4 shows the position of emission bands of (5 and 10% Cu doped ZnS/Fe_3_O_4_) nanocomposites with just a modest shift in PL spectra.

**Table 4:** PL bands position in (5 and 10 wt%) Cu doped ZnS/Fe_3_O_4_.

Sample	PL bands position (nm)		
5 wt% Cu doped ZnS/Fe_3_O_4_	421	483	513
10 wt% Cu doped ZnS/Fe_3_O_4_	424	481	517

### UV–Visible (UV–Vis) spectroscopy study

**Figure 5:**
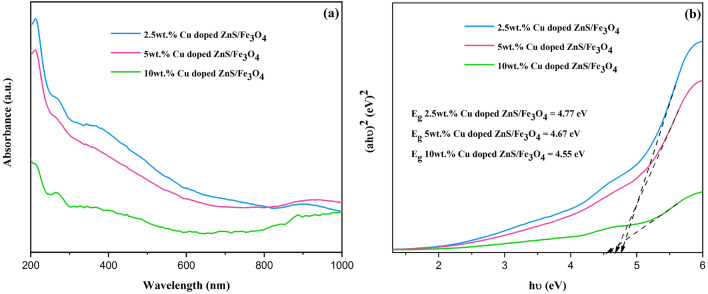
(**a**) UV–Vis spectra and (**b**) plots of ($\alpha h\upsilon )$^2^ vs. photon energy ($h\upsilon$) for the bandgap energy of (2.5, 5 and 10 wt%) Cu doped ZnS/Fe_3_O_4_ nanocomposites.

Utilizing a UV–Vis spectrometer, the optical characteristics of the nanocomposites were examined. Figure 5a,b show the absorbance spectra and their corresponding estimated band gaps. Because of their absorption, the synthesized samples are acceptable materials for the treatment of wastewater. For 2.5 wt% Cu, 5 wt% Cu and 10 wt% Cu doped ZnS/Fe_3_O_4_, the absorption band emerged at 212, 211, and 264 nm, respectively. The bandgap value alternates as the Cu concentrations change. For 2.5 wt% Cu, 5 wt% Cu and 10 wt% Cu, the computed band values are 4.77, 4.67, and 4.55 eV, respectively. The closing in the bandgap values is a result of fluctuating carrier concentrations, which arise from the introduction of defects, leading to the formation of new energy levels between the valence and conduction bands in the samples^42^. In comparison to other samples, the band gap energy is smaller in 10 wt% Cu doped ZnS/Fe_3_O_4_. This means that more e^−^/h^+^ couples may be generated by UV light energy and more separation rate of charge carriers, which increases photocatalytic performance^51^.

### Electronic structure study

**Figure 6:**
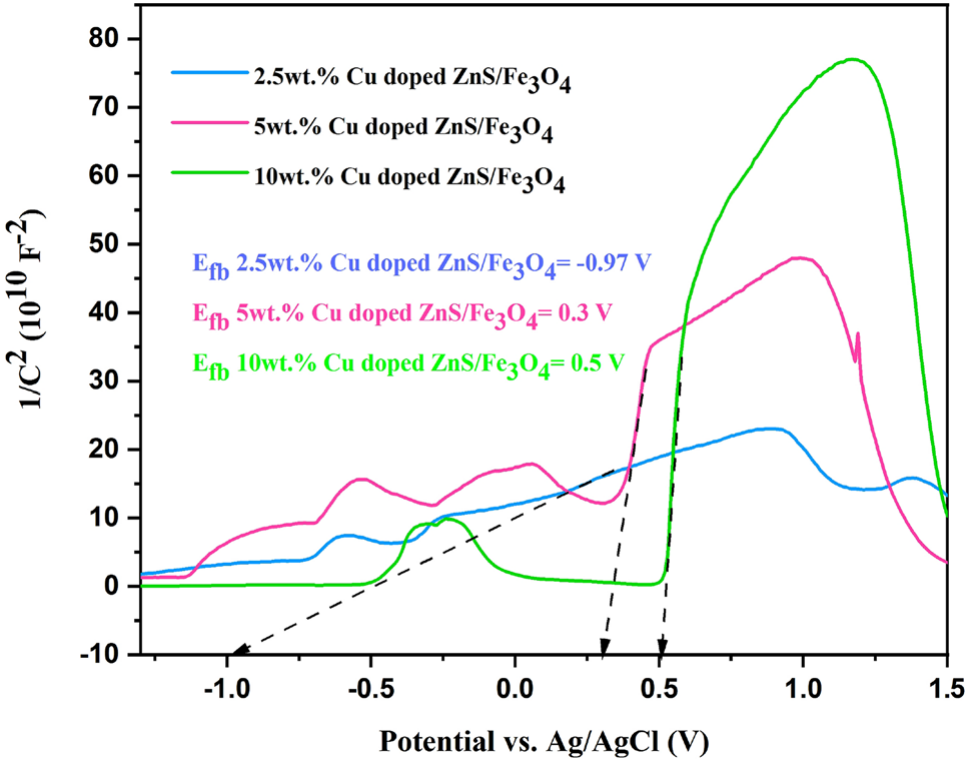
Mott–Schottky curves of the (2.5, 5 and 10 wt% Cu) doped ZnS/Fe_3_O_4_.

Mott-Schottky analysis is commonly employed in electrochemical analysis to measure the charge-current density and flat band potential (Efb), and also to analyze the electronic behavior of various materials, including n-type/p-type/p-n type^52^. The Mott–Schottky (M–S) test, as depicted in Fig. 6, was conducted to determine the precise locations of the conduction band (CB) and valence band (VB) of synthesized samples. The positive slopes observed in the linear portions of the MS curves signifying that the 2.5, 5, and 10 are n-types semiconductors^53^. Additionally, the slope of the Mott–Schottky plot and its related intercept with the x-axis were calculated for each sample in order to determine the carrier concentration and flat band potential, respectively (as shown in Table 5)^54^. According to data analysis, 10 wt% Cu-doped ZnS/Fe_3_O_4_ has the highest and 2.5 wt% Cu doped ZnS/Fe_3_O_4_ has the lowest carrier concentration. The 10 wt% Cu doped ZnS/Fe_3_O_4_ nanocomposite’s electrical resistance may be significantly reduced as a result of the enormous increase of charge carriers. This result is in excellent agreement with the outcomes of PL investigations and the successful separation of charges. Furthermore, the 10 wt% Cu doped ZnS/Fe_3_O_4_ shows the largest flat band potential (0.5 V), indicating that a smaller overpotential can be used to start the charge-transfer reaction^54^. The Ag/AgCl reference electrode was used to calculate the samples’ flat band potentials. Based on the observation, the flat-band potential (EFB) of n-type semiconductors was generally near the CB position, the estimated CB of (2.5, 5, and 10 wt%) Cu doped ZnS/Fe_3_O_4_ were − 0.873, 0.397 and 0.597 eV (NHE), respectively. The calculated values of the VB were 3.89, 5.07, and 5.15 eV, respectively, based on the formula E_CB_ = E_VB_ − E_g_^55^.

**Table 5:** Carrier concentrations (N_D_) and flat band potentials of the (2.5, 5 and 10 wt% Cu) doped ZnS/Fe_3_O_4_.

Sample	Carrier concentration (cm^−3^)	Flat band potential (V)
2.5wt.% Cu doped ZnS/Fe_3_O_4_	6.97 × 10^10^	− 0.97
5wt.% Cu doped ZnS/Fe_3_O_4_	1.15 × 10^11^	0.3
10wt.% Cu doped ZnS/Fe_3_O_4_	2.47 × 10^11^	0.5

### Morphological and compositional (FE-SEM & EDX) study

**Figure 7:**
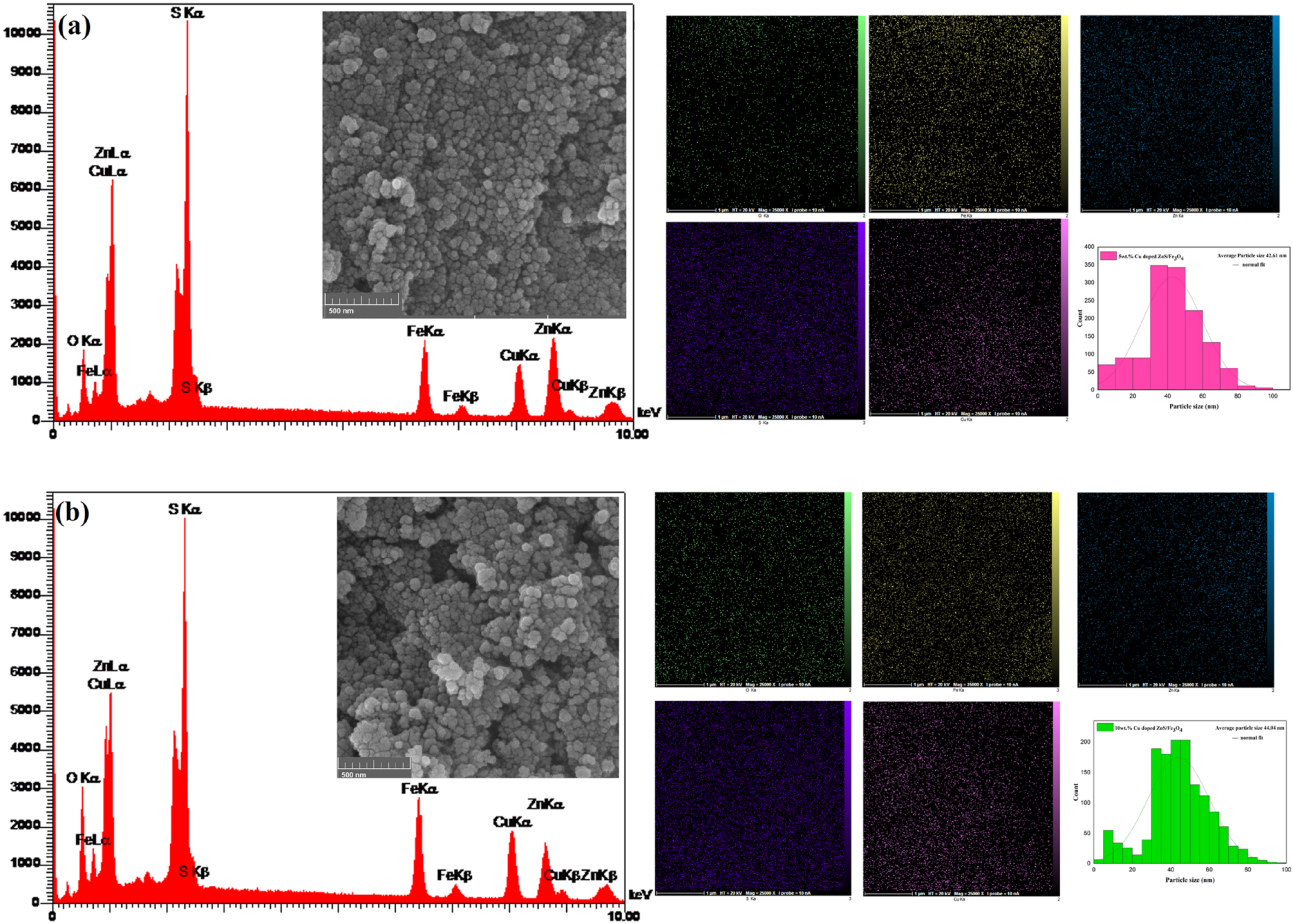
FESEM images, EDS patterns, EDS elemental mappings (Fe (yellow dots), O (green dots), Zn (blue dots), S (purple dots) and Cu (red violet dots)) and particle size distribution of (**a**) 5 wt% Cu doped ZnS/Fe_3_O_4_ and (**b**) 10 wt% Cu doped ZnS/Fe_3_O_4_ nanocomposites.

Structural and compositional examination of the synthesized samples has been carried out using the FE-SEM and EDS method, as depicted in Fig. 7a,b. The FE-SEM images of the (5 and 10 wt%) Cu-doped ZnS/Fe_3_O_4_ nanocomposites, reveal a uniform and symmetrical spherical morphology with some degree of agglomeration. No discernible variations in morphology were observed between the 5 wt% Cu doped ZnS/Fe_3_O_4_ and 10 wt% Cu doped ZnS/Fe_3_O_4_. The average size of the particles in the 5 wt% Cu doped ZnS/Fe_3_O_4_ and 10 wt% Cu doped ZnS/Fe_3_O_4_ nanocomposites is 42.61 and 44.04 nm, respectively. In addition, the elemental mapping of nanocomposites elucidates the dispersion of copper, iron, oxygen, zinc, and sulfur elements. The presence of Cu, Fe, O, Zn, and S elements is confirmed by the EDS spectra of the Cu doped ZnS/Fe_3_O_4_ samples, without any impurities, which is consistent with the XRD results. The synthesized materials are devoid of impurities and residual chemicals, thereby ensuring their safety for photocatalytic applications. The weight and atomic percentage of elements in synthesized materials showed in Table 6.

**Table 6:** EDS quantification results.

Element	Weight (%)		Atomic (%)	
	5wt% Cu doped ZnS/Fe_3_O_4_	10wt% Cu doped ZnS/Fe_3_O_4_	5wt% Cu doped ZnS/Fe_3_O_4_	10wt% Cu doped ZnS/Fe_3_O_4_
O	17.30	23.29	30.88	33.94
S	45.32	40.38	45.60	38.01
Fe	13.06	14.20	11.39	17.67
Cu	7.58	11.92	3.86	5.66
Zn	16.74	10.21	8.27	4.72
Total	100	100	100	100

### Adsorption ability of (2.5, 5 and 10 wt%) Cu doped ZnS/Fe_3_O_4_

**Figure 8:**
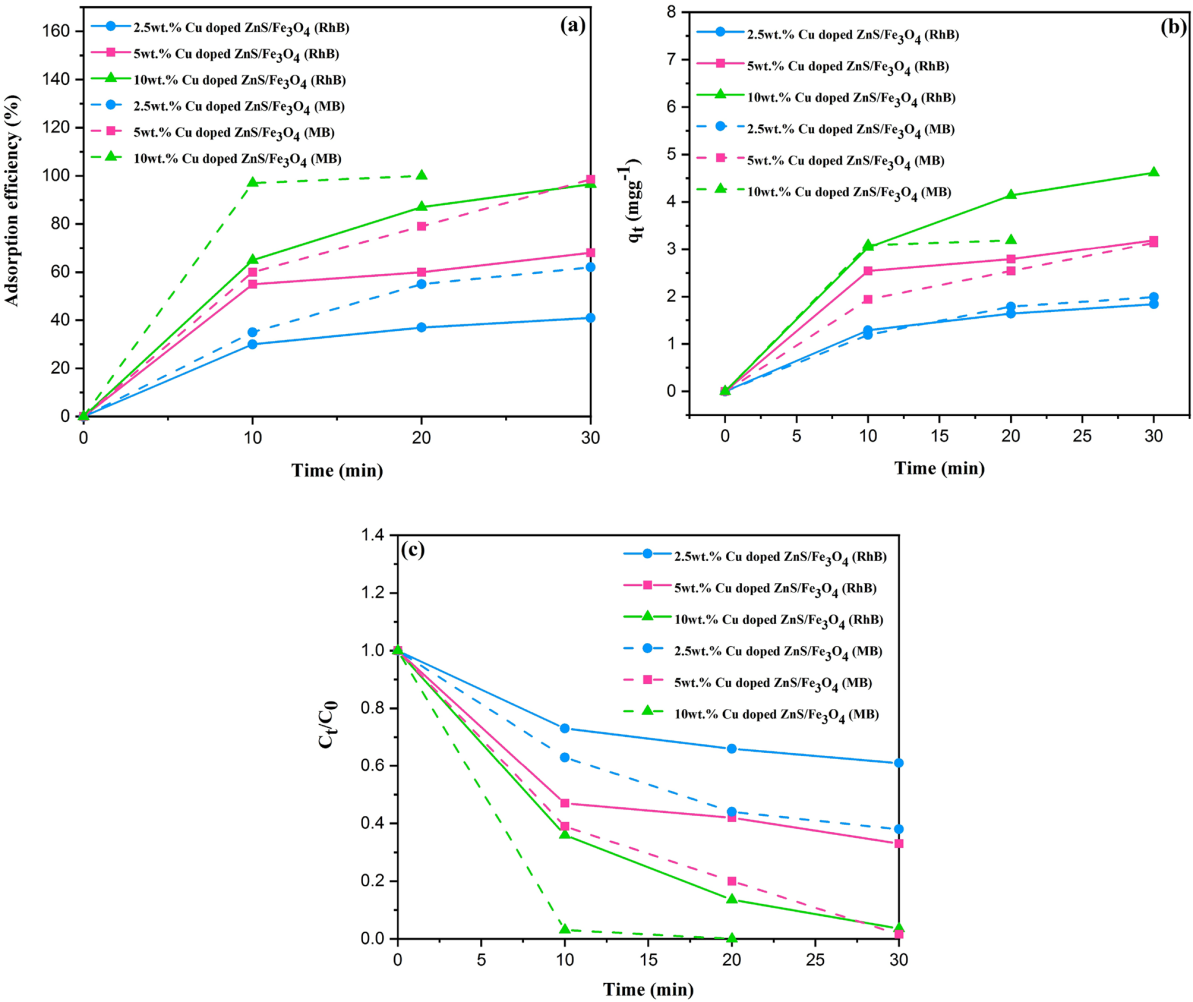
(**a**) Adsorption efficiency, (**b**) adsorption capacity and (**c**) C_t_/C_0_ plots of 2.5, 5 and 10wt% Cu doped ZnS/Fe_3_O_4_.

To evaluate the adsorption abilities of (2.5, 5 and 10 wt%) Cu-doped ZnS/Fe_3_O_4_, RhB and MB have been selected as the organic dyes. The impact of time on the adsorption efficiency was examined through the utilization of UV–Vis spectra (Fig. 8a). As illustrated, the quantity of dye adsorbed onto the photocatalysts from aqueous solution grows rapidly over time. Furthermore, an investigation of adsorption efficiency in various samples reveals that, 10 wt% Cu doped ZnS/Fe_3_O_4_ has the maximum adsorption efficiency for MB and RhB, with 100% of MB adsorbed in 20 min. For RhB, the equilibrium adsorption capacity of (2.5, 5, and 10 wt%) Cu doped ZnS/Fe_3_O_4_ is 1.84 mg g^−1^, 3.19 mg g^−1^, and 4.615 mg g^−1^, respectively and for MB the equilibrium adsorption capacity of (2.5 and 5 wt%) Cu doped ZnS/Fe_3_O_4_ is 1.99 mg g^−1^ and 3.14 mg g^−1^, respectively (Fig. 8b). As shown in Fig. 8c, these results corresponded to the C_t_/C_0_ plots of (2.5, 5, and 10 wt%) Cu doped ZnS/Fe_3_O_4_.

### Kinetics of RhB degradation

The Langmuir–Hinshelwood (LH) model is commonly employed to elucidate the kinetics of dye degradation. This model highlights that RhB degradation exhibits conformity with the pseudo-first order kinetics at low concentrations. Consequently, the data underwent scrutiny to assess their compatibility with the pseudo-first order kinetics using the subsequent formula^56^.

4 $$\text{ln}(\frac{{C}_{0}}{{C}_{t}})=-Kt.$$

K represents the pseudo-first-order rate constant (min^−1^). C_0_ and C_t_ denote the first and final concentration of the RhB dye at any radiation time (mg L^−1^) and t is the duration of irradiation (min)^56–58^.

**Figure 9:**
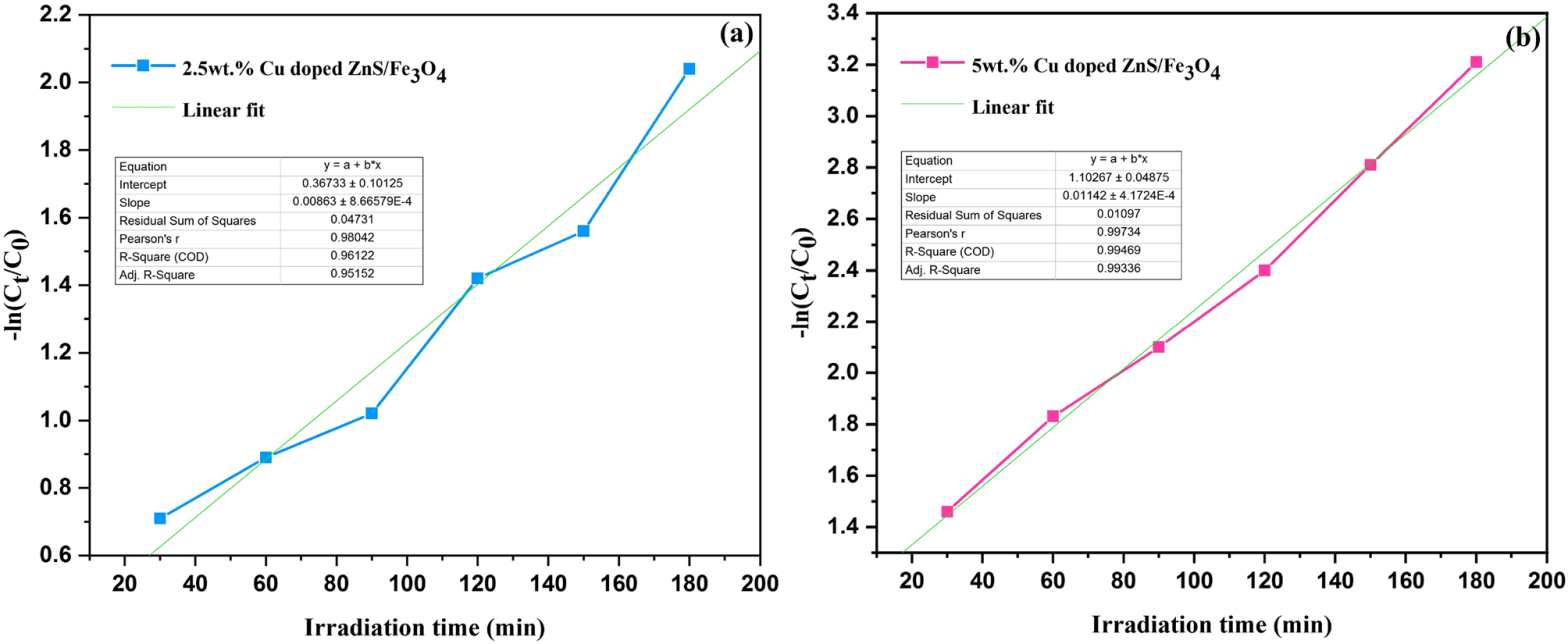
Plot of − ln (C_t_/C_0_) vs. irradiation time for (**a**) 2.5 wt% Cu doped ZnS/Fe_3_O_4_ and (**b**) 5 wt% Cu doped ZnS/Fe_3_O_4_ under UV light.

Figure 9 (− ln(C_0_/C_t_) vs. time) exhibits a linear trend for RhB degradation with the use of (2.5 and 5 wt%) Cu doped ZnS/Fe_3_O_4_. The computed rate constant from the kinetics plot and the related correlation coefficients (R^2^) are shown in Table 7. As demonstrated, the 5 wt% Cu doped ZnS/Fe_3_O_4_ has a higher apparent rate constant than 2.5 wt% Cu doped ZnS/Fe_3_O_4_. Consequently, 5 wt% Cu doped ZnS/Fe_3_O_4_ exhibits superior photocatalytic performance than 2.5 wt% Cu doped ZnS/Fe_3_O_4_ for the degradation of RhB dye.

**Table 7:** Pseudo first-order rate constants and related R^2^ values of (2.5 and 5 wt%) Cu doped ZnS/Fe_3_O_4_ under UV light.

Sample	Rate constant (K) (min^−1^)	R^2^
2.5 wt% Cu doped ZnS/Fe_3_O_4_	0.0086	0.9612
5 wt% Cu doped ZnS/Fe_3_O_4_	0.0114	0.9947

### Photocatalytic mechanism and degradation study

When photocatalysis process occurs, the dye molecules react with electron–hole pairs at the conduction and valence bands, resulting in superoxide radicals (^⋅^O_2_^–^) and hydroxyl radicals (^⋅^OH), upon exposure to light of an energy level surpassing the prepared sample’s bandgap. The degradation of dyes such as RhB, resulting in the formation of H_2_O and CO_2_, can be attributed to radicals such as superoxide, and hydroxyl. Another factor contributing to the enhancement of photocatalytic activity is the generation of a novel energy level situated beneath the conduction band, which occurs as the dopant concentration increases^42^.

**Figure 10:**
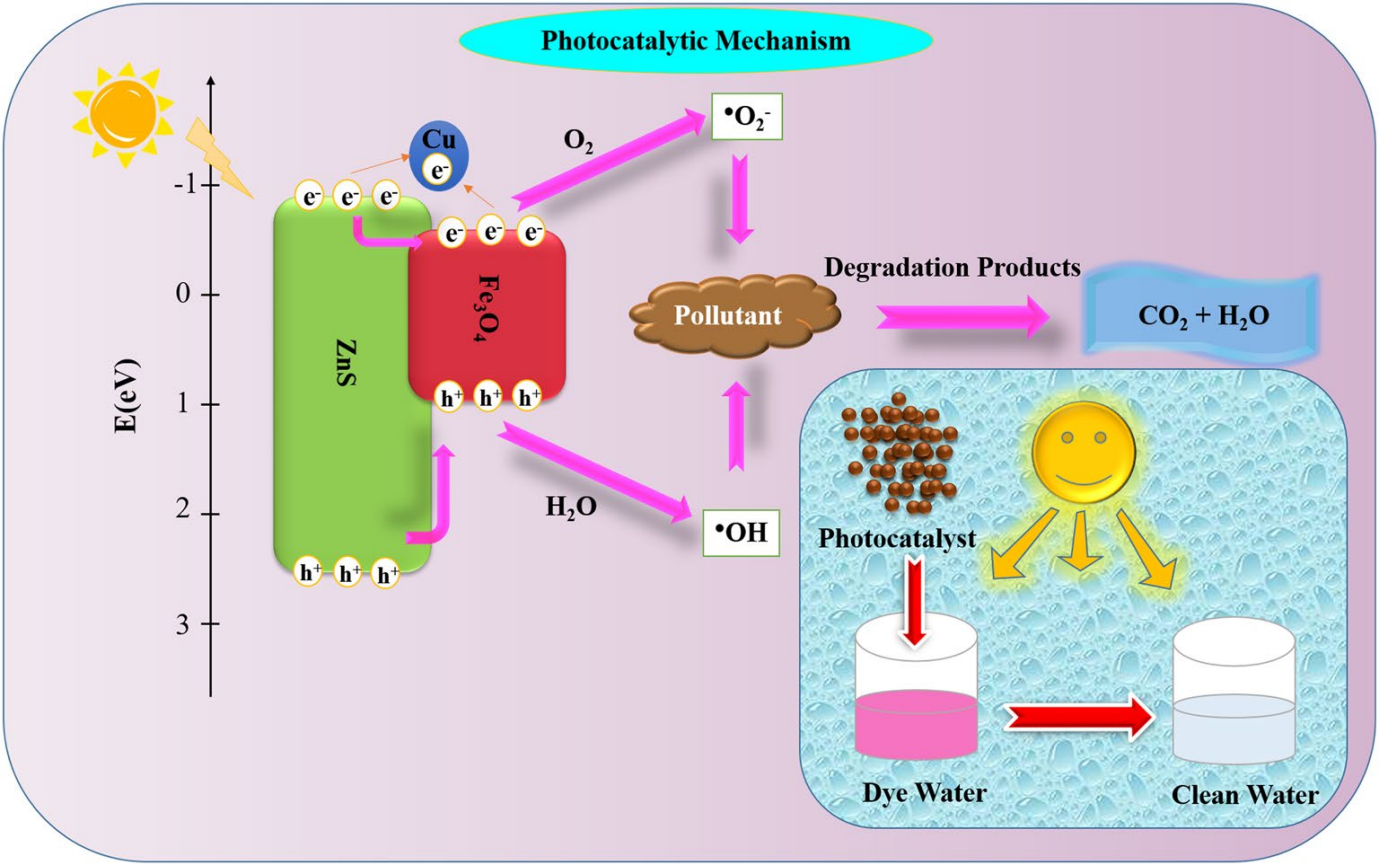
Proposed photocatalytic mechanism of Cu doped ZnS/Fe_3_O_4_.

The Cu doped ZnS/Fe_3_O_4_ photocatalytic mechanism is proposed and presented in Fig. 10. Electrons from the valence band are excited by UV light and subsequently transferred to the Cu^2+^ level, resulting in the formation of Cu^1+^ ions. The Cu^1+^ ions have the ability to decrease the adsorbed molecular oxygen (O_2_) and convert it into superoxide radicals. Additionally, the valence band of ZnS/Fe_3_O_4_ generates holes that interact with water molecules and ultimately produce hydroxyl radicals (^⋅^OH). These radicals can select organic pollutants on or near the photocatalyst’s surface through oxidation or reduction processes^59^. The dye photodegradation reactions can be described as follows:

5 $$ {\text{h}}\upsilon \, + {\text{ ZnS}}/{\text{Fe}}_{{3}} {\text{O}}_{{4}} \to {\text{e}}^{ - } + {\text{ h}}^{ + } , $$

6 $$ {\text{e}}^{ - } + {\text{ Cu}}^{{{2} + }} \to {\text{Cu}}^{ + } , $$

7 $$ {\text{Cu}}^{ + } + {\text{ O}}_{{2}} \to^{ \cdot } {\text{O}}_{{2}}^{ - } , $$

8 $$ {\text{h}}^{ + } + {\text{ H}}_{{2}} {\text{O}} \to^{ \cdot } {\text{OH,}} $$

9 $$ {\text{e}}^{ - } + {\text{ O}}_{{2}} \to^{ \cdot } {\text{O}}_{{2}}^{ - } , $$

10 $$^{ \cdot } {\text{OH }} + {\text{ Pollutant}} \to {\text{CO}}_{{2}} + {\text{ H}}_{{2}} {\text{O,}} $$

11 $$^{ \cdot } {\text{O}}_{{2}}^{ - } + {\text{ Pollutant}} \to {\text{CO}}_{{2}} + {\text{ H}}_{{2}} {\text{O}}{.} $$

**Figure 11:**
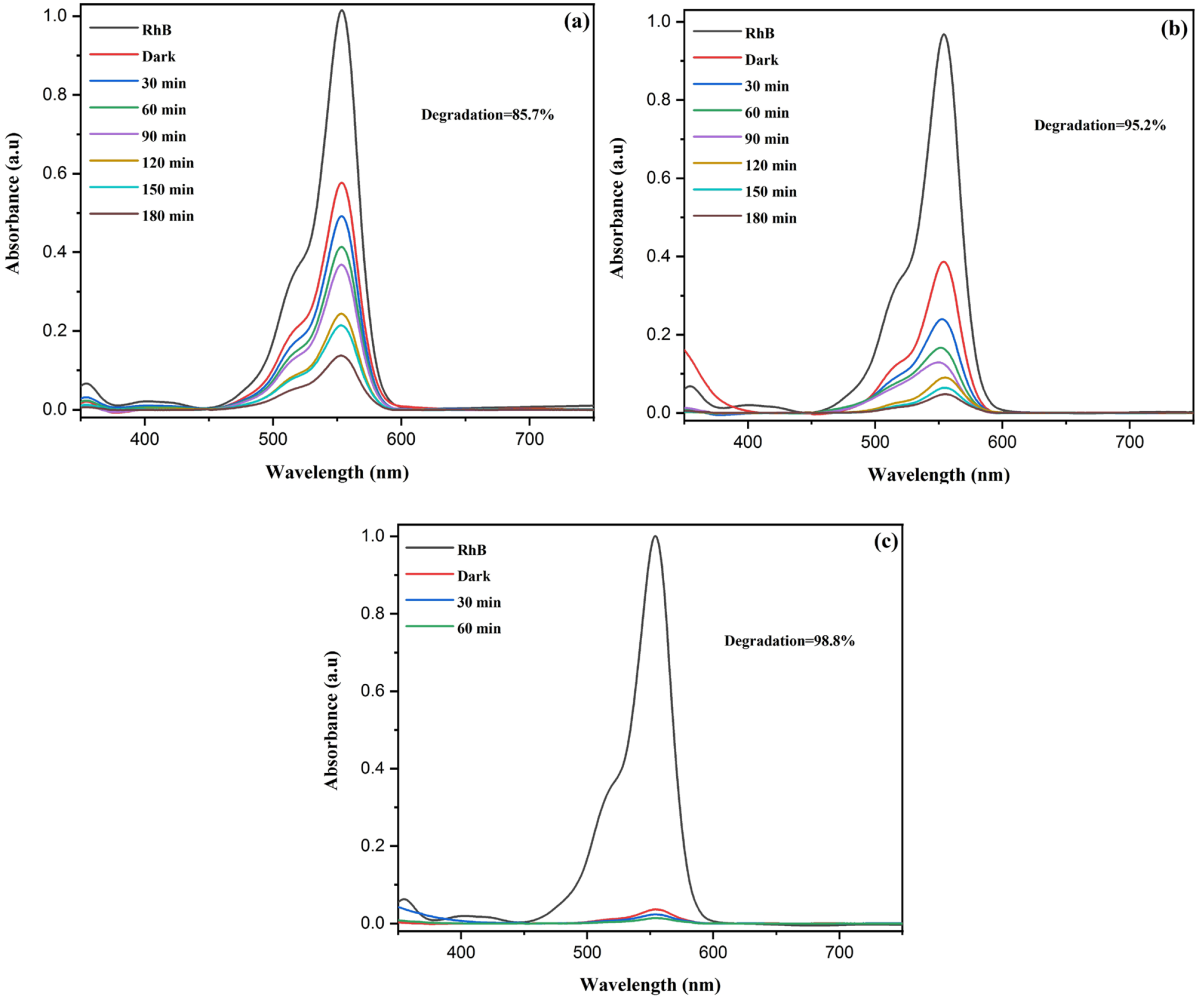
UV–Vis absorption spectra of RhB dye solution at different reaction times in the presence of (**a**) 2.5 wt% Cu, (**b**) 5 wt% Cu and (**c**) 10 wt% Cu doped ZnS/Fe_3_O_4_.

The assessment of the photocatalytic efficacy of fabricated samples is conducted through the utilization of rhodamine B (RhB). Figure 11 show the UV spectrum of RhB dye in relation to reaction time, effectively demonstrating the photocatalytic capabilities of the synthesized samples in degradation of RhB dye. Before irradiation, the dye solutions accompanied by a catalyst, were placed in a dark environment (30 min), to attain adsorption–desorption equilibrium. For 180 min, the RhB dye solution was exposed to UV light. After a uniform 30 min interval, the aqueous dye solution was removed and subjected to UV–Vis spectroscopy for characterization. Figure 11 illustrates the alteration of the absorbance peak at 554 nm with an increase in the concentration of dopant from 2.5 to 10 wt%. As absorption reduces with respect to illumination time, the degradation efficiency rises. At the same degradation situations applied to the all prepared samples, the 10 wt% Cu had the greatest reduction in the absorption peak.

After 60 min of irradiation, 10 wt% Cu doped ZnS/Fe_3_O_4_ had the most degradation efficiency (i.e. 98.8%). With the assistance of PL spectroscopy information, which demonstrates that the sample with the smallest intensity in PL spectra has stronger photocatalysis degradation, this highest efficiency and effectiveness of the 10 wt% Cu doped ZnS/Fe_3_O_4_ may be thoroughly substantiated. As can be observed, the sample containing 10 wt% Cu has larger degrading activity due to its lower PL spectrum intensity.

### Reusability of recycled photocatalysts

**Figure 12:**
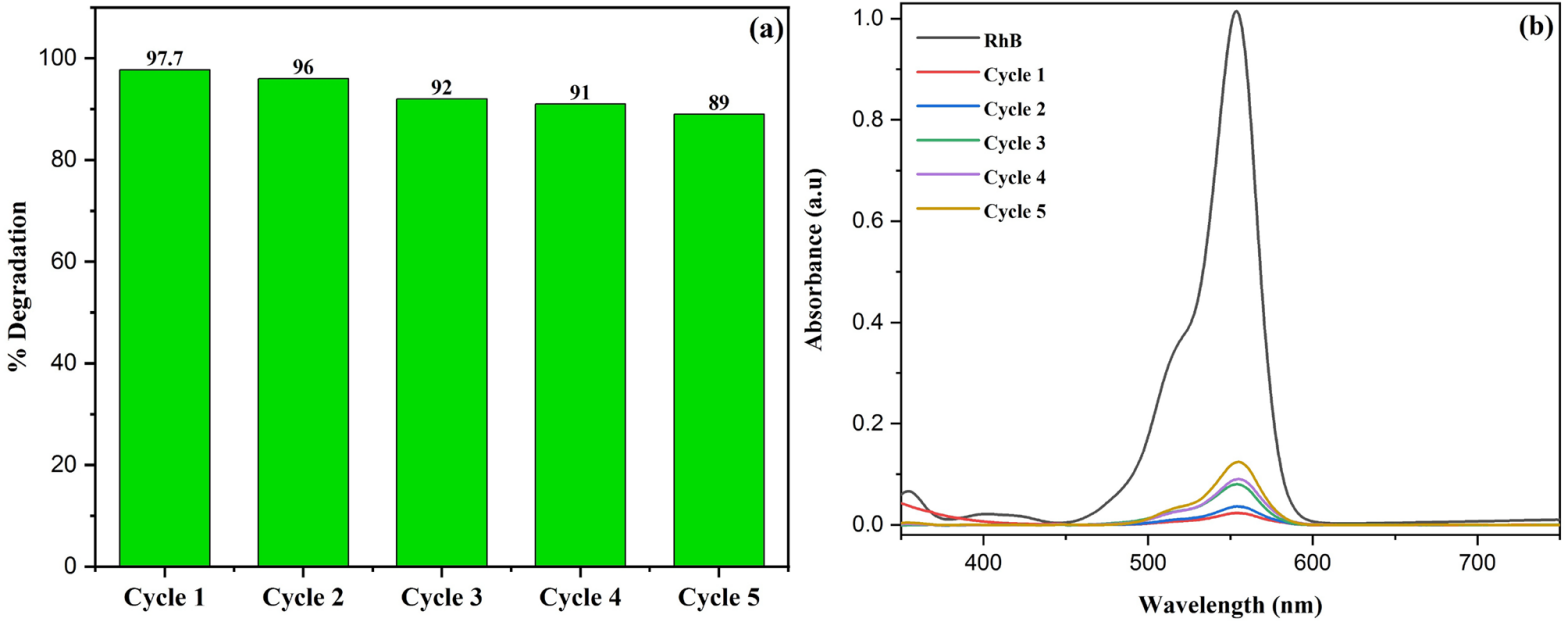
(**a**) Cyclic photocatalysis and (**b**) corresponding UV absorbance spectra of 10 wt% Cu doped ZnS/Fe_3_O_4_ nanocomposite.

The photocatalytic process is typically conducted within a suspension comprised of semiconductor nanostructures, necessitating a subsequent separation procedure to eliminate the catalyst from the aqueous medium. The cost incurred in eliminating nanoparticles, from substantial quantities of water, poses a significant impediment to the photocatalytic technique employed for wastewater treatment^60^. However, the nanocomposites generated in this investigation show superparamagnetic characteristics at room temperature, which simplifies the previously discussed problem. When creating photocatalysts for industrial applications, reusability is of the utmost importance^61^. In the present study, an investigation was conducted to assess the reusability of 10 wt% Cu doped ZnS/Fe_3_O_4_. The photocatalyst underwent a sequence of procedures involving continuous testing, recycling through the use of a magnet, washing, drying, and subsequent reuse for a total of five cycles. The outcomes of these experiments are illustrated in Fig. 12. The degradation efficiency gradually decreased from the first to the fifth cycles, exhibiting values of 97.7%, 96%, 92%, 91%, and 89%, respectively. Since 100% recovery of photocatalyst is not attainable, the decline may be the result of catalyst loss in the recovering operations or obstruction of some active sites by dye adsorption that cannot be totally eliminated in each cycle by ethanol or water^62^. The findings indicate that after five cycles, 89% of the photocatalytic activity of 10 wt% Cu doped ZnS/Fe_3_O_4_ may be sustained without a discernible decrease.

**Figure 13:**
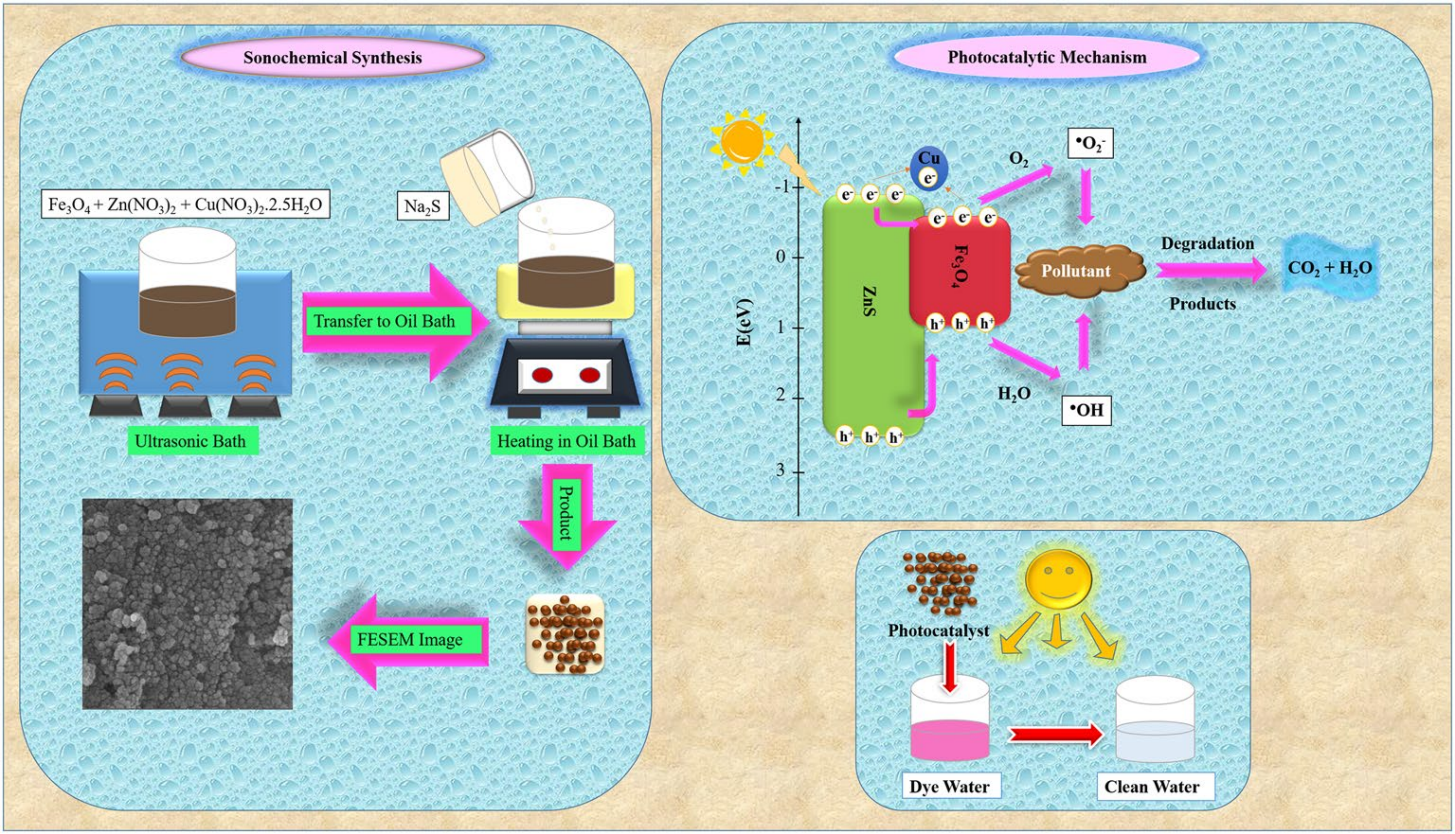
Illustration of synthesis procedure of Cu doped ZnS/Fe_3_O_4_ nanocomposite along with FE-SEM image and photocatalytic mechanism in degradation of wastewater.

Overall, Fig. 13 presents the synthesis procedure of Cu doped ZnS/Fe_3_O_4_ nanocomposite, complemented by an FE-SEM image. Additionally, it provides an elucidation of the photocatalytic mechanism employed in the degradation of wastewater.

## Conclusions

The release of dye wastewater by many industries endangers the environment and human health. Dye photodegradation emerges as a viable method for industrial wastewater treatment, providing an environmentally acceptable and cost-effective alternative. We created Cu doped ZnS/Fe_3_O_4_ nanocomposites using a rapid sonochemical technique, demonstrating their adaptability for both adsorption and photocatalytic degradation of organic pollutants, and the 10 wt% Cu doped ZnS/Fe_3_O_4_ displayed outstanding photostability and reusability by magnetic separation. These samples demonstrated exceptional adsorption capability in the decolorization of several dyes (RhB and MB). Adsorption capacity in various samples shows that, the highest adsorption efficiency for MB and RhB is found in 10 wt% Cu doped ZnS/Fe_3_O_4_, which 100% MB adsorbed in 20 min. Also, the 10 wt% Cu doped ZnS/Fe_3_O_4_ had the highest reduction in the absorption peak when the same degradation conditions were applied to all prepared samples. It exhibited the highest degradation efficiency (98.8%) after 60 min of UV irradiation. This sample revealed remarkable recyclability, maintaining a degradation rate of 89% following five cycles. The findings highlight that the synthesized samples show the potential of photocatalytic approach as a feasible and eco-friendly technique for remedying industrial wastewater that is polluted with dyes.

## Supplementary Information

Supplementary Information.

## Data Availability

All data generated or analysed during this study are included in this published article [and its Supplementary Information files].
